# Supplementary material for: Prevalence and Associated Factors of HPV Infection in the Oropharyngeal Cavity Among University Students in a Southwest Population in Mexico
Source: Diseases. 2025 Dec 31;14(1):16. doi: 10.3390/diseases14010016 (PMC12840128; doi:10.3390/diseases14010016)
Supplement: Supplementary file 1 [file diseases-14-00016-s001.zip › CONBIOETICA register.pdf]

CENTRO DE ALTA ESPECIALIDAD DEL ESTADO DE VERACRUZ "DR. RAFAEL LUCIO"

SS Secretaría de Salud | SESVER Servicios de Salud de Veracruz

SUBDIRECCIÓN DE ENSEÑANZA
DEPARTAMENTO DE INVESTIGACIÓN

CAE

CÉDULA DE EVALUACIÓN DEL COMITÉ DE INVESTIGACIÓN

Registro Autorización COFEPRIS 19 CI 30 087 028

EN LA CIUDAD DE XALAPA, VERACRUZ, SIENDO LAS 11:00 HORAS DEL DÍA 29 MAYO 2023 ESTANDO REUNIDOS LOS INTEGRANTES DEL COMITÉ DE INVESTIGACIÓN, EN LA SALA DE JUNTAS DE LA UNIDAD DE INVESTIGACIÓN DE ESTA INSTITUCIÓN, SE PROCEDIÓ A REALIZAR LA REVISIÓN DEL PROTOCOLO DE INVESTIGACIÓN TITULADO: **"PREVALENCIA Y FACTORES ASOCIADOS A INFECCIÓN POR VPH EN CAVIDAD OROFARÍNGEA EN ESTUDIANTES UNIVERSITARIOS".** QUE PRESENTA LA C. DANIELA CÓRDOBA COLORADO, ESTANDO PRESENTES: DR. JOSÉ GERARDO ALONSO ACOSTA, MASS. MA. MARTHA GÓMEZ LANDERO TELLO, MATRA. EUGENIA SACRAMENTO MUNDO Y DR. JUAN GERARDO NEME KURI.

FUERON REVISADOS Y ACEPTADOS LOS SIGUIENTES RUBROS:

CARACTERÍSTICAS		
**SE ACEPTO:**	**SI**	**NO**
PORTADA	(X)	( )
INDICE	(X)	( )
ABREVIATURAS	(X)	( )
RESUMEN*	(X)	( )
INTRODUCCIÓN	(X)	( )
1. ANTECEDENTES	(X)	( )
2. PLANTEAMIENTO	(X)	( )
3. JUSTIFICACIÓN	(X)	( )
4. OBJETIVOS	(X)	( )
4.1 Objetivo General	(X)	( )
4.2 Objetivos Específicos	(X)	( )
5. HIPÓTESIS	(X)	( )
6. METODOLOGÍA	(X)	( )
6.1 Tipo de estudio o diseño	(X)	( )
6.2 Población Objetivo	(X)	( )
6.2.1 Criterios de inclusión	(X)	( )
6.2.2 Criterios de exclusión	(X)	( )
6.2.3 Criterios de eliminación	(X)	( )

*Cumple con el requisito administrativo del Registro Nacional de Proyectos de Investigación (RENAPI) de la DGCES.

CARACTERÍSTICAS		
**SE ACEPTO:**	**SI**	**NO**
6.3 Ubicación espacio-temporal	(X)	( )
6.4 Definición Operacional	(X)	( )
6.5 Unidad o Sujeto Estudio	(X)	( )
6.6 Obtención de unidades	(X)	( )
6.7 Factores de confusión	(X)	( )
6.8 Tabla de variables	(X)	( )
6.9 Descripción del proyecto	(X)	( )
6.10 Tamaño de muestra	(X)	( )
7. ANÁLISIS ESTADÍSTICO	(X)	( )
8. RECURSOS	(X)	( )
8.1 Recursos Humanos	(X)	( )
8.2 Recursos Materiales	(X)	( )
8.3 Recursos Financieros	(X)	( )
9. ÉTICA**	(X)	( )
10. LOGÍSTICA	(X)	( )
11. REFERENCIAS	(X)	( )
12. ANEXOS	(X)	( )

**Cumple con el procedimiento administrativo de la Coordinación de Investigación.

ANOTE AL REVERSO LAS OBSERVACIONES DE LOS RUBROS NO ACEPTADOS

EL COMITÉ EMITIÓ EL SIGUIENTE DICTAMEN: **APROBADO FOLIO 28/23**

OTROS ASUNTOS: NINGUNO

FIRMAN AL CALCE LOS PRESENTES A LAS 12:00 HORAS DEL MISMO DÍA 29 DE MAYO 2023

DR. JUAN GERARDO NEME KURI
PRESIDENTE

DR. JOEL JAHAZIEL DÍAZ VALLEJO
SECRETARIO

MTRA. EUGENIA SACRAMENTO MUNDO
VOCAL

MASS. MA MARTHA GÓMEZ LANDERO TELLO
VOCAL

DR. JOSÉ GERARDO ALONSO ACOSTA
VOCAL

Centro de Alta Especialidad "Dr. Rafael Lucio"
COMITÉ DE INVESTIGACIÓN
Autorización COFEPRIS 19 CI 30 087 028
Xalapa, Ver.

FR-CI-06
Versión: 2.

ACTA DEL COMITÉ DE ÉTICA EN INVESTIGACIÓN

Registro Autorización CONBIOÉTICA-30-CEI-001-20170221

C. Daniela Córdoba Colorado
Estudiante de Licenciatura en QFB
Universidad Veracruzana
Circuito Gonzálo Aguirre Beltrán, Esq Calle de la Pergola
Zona Universitaria, Xalapa-Enríquez, Ver.

El Comité de Ética en Investigación del Centro de Alta Especialidad "Dr. Rafael Lucio", con relación al estudio de investigación, Titulado: **"Prevalencia y factores asociados a infección por VPH en cavidad orofaríngea en estudiantes universitarios"**, se ha revisado y aprobado bajo el número de folio 28/23.

ATENTAMENTE

Mtro. Sergio Arturo González Ortiz
PRESIDENTE
COMITÉ DE ÉTICA EN INVESTIGACIÓN

Centro de Alta Especialidad
"Dr. Rafael Lucio"
COMITÉ DE ÉTICA EN INVESTIGACIÓN
Autorización CONBIOÉTICA
30 CEI 001 20170221
Xalapa, Ver.
