# Supplementary material for: Prevalence and Associated Factors of HPV Infection in the Oropharyngeal Cavity Among University Students in a Southwest Population in Mexico
Source: Diseases. 2025 Dec 31;14(1):16. doi: 10.3390/diseases14010016 (PMC12840128; doi:10.3390/diseases14010016)
Supplement: Supplementary file 1 [file diseases-14-00016-s001.zip › Conflict of interest Declaration Form.pdf]

Conflict of interest Declaration Form

The authors have no conflicts of interest to declare.
All co-authors have seen and agree with the contents of the manuscript and there is no financial interest to report. We certify that the submission is original work and is not under review at any other publication.

1. Name: Prof. Joel J. Díaz

Signature: Date: 30 / 09 / 2025

2. Name: Miss. Daniela Córdoba

Signature: Date: 30 / 09 / 2025

3. Name: Miss. Dulce del C. González

Signature: Date: 30 / 09 / 2025

4. Name: Ezri Cruz

Signature: Date: 30/09/2025

5. Name: Magda O. Pérez

Signature: Date: 30 / 09 / 2025

6. Name: José Locia

Signature: Date: 30 / 09 / 2025

7. Name: Luz I. Pascual

Signature: Date: 30 / 09 / 2025
