# Supplementary material for: Prevalence and Associated Factors of HPV Infection in the Oropharyngeal Cavity Among University Students in a Southwest Population in Mexico
Source: Diseases. 2025 Dec 31;14(1):16. doi: 10.3390/diseases14010016 (PMC12840128; doi:10.3390/diseases14010016)
Supplement: Supplementary file 1 [file diseases-14-00016-s001.zip › Letters.pdf]

HOJA INFORMATIVA PARA PARTICIPAR EN EL ESTUDIO DE INVESTIGACIÓN:

“Prevalencia y factores asociados a infección por VPH en cavidad orofaríngea en estudiantes universitarios”.

Que consiste en estimar la prevalencia de infección por VPH en cavidad orofaríngea en una población estudiantil sexualmente activa, para lo cual se me realizará una breve encuesta confidencial y se me tomará una muestra bucal NO INVASIVA e INDOLORA que será almacenada en un recipiente estéril y transportada al laboratorio de Biología Molecular Aplicada para su análisis.

Sobre mi participación en el estudio:

Mi participación en este estudio es totalmente voluntaria y confidencial. Si acepto participar, pero decido retirarme, puedo hacerlo con libertad en cualquier momento y no pasará nada.

Sobre mi privacidad en el estudio:

Mis datos personales no serán difundidos por ningún medio de acuerdo con las leyes ARCO, respetando en todo momento mi privacidad, por lo que sólo el equipo investigador tendrá acceso a la información proporcionada, recabada con ayuda de una encuesta.

Este estudio se llevará a cabo según la normativa ética vigente (Declaración de Helsinki y a la Norma Oficial Mexicana NOM-012-SSA3-2012, que establece los criterios para la ejecución de proyectos de investigación para la salud en seres humanos). Es muy importante comprender que la encuesta que voy a responder contiene datos personales muy sensibles (se preguntará sobre hábitos sexuales), ésta encuesta será completamente confidencial, es decir, se me asignará un número para identificarla una vez respondida, y éste mismo número se le asignará a mi muestra biológica, así se evitará colocar mi nombre. Debo saber que el procedimiento será extremadamente cuidadoso, por lo que se tomarán todas las medidas para que nadie relacione mi nombre con la encuesta. Utilizándose los datos únicamente con fines académicos y de investigación.

Si existe alguna duda con respecto al estudio o a la confidencialidad de los datos, puedo consultar con los investigadores a cargo de este estudio, la Dra. Luz Irene Pascual Mathey, y el Dr. Joel Jahaziel Díaz Vallejo, o con la estudiante tesista encargada de este estudio, Daniela Córdoba Colorado, a través del correo electrónico: proyectoprevalenciavph@gmail.com

EN TÉRMINOS DE LA LEY 316 DE PROTECCIÓN DE DATOS PERSONALES EN POSESIÓN DE SUJETOS OBLIGADOS PARA EL ESTADO DE VERACRUZ DE IGNACIO DE LA LLAVE, SE LES INFORMA QUE LAS PERSONAS A CARGO DEL PROYECTO SON RESPONSABLES DEL USO Y TRATAMIENTO QUE SE LE DÉ A LA INFORMACIÓN PERSONAL PROPORCIONADA POR USTED, ASÍ COMO DE SU PROTECCIÓN, COMPROMETIÉNDOSE EN TODO MOMENTO A QUE ESTA SERÁ TRATADA BAJO LAS MÁS ESTRICTAS MEDIDAS DE SEGURIDAD QUE GARANTICEN SU CONFIDENCIALIDAD, INTEGRIDAD Y DISPONIBILIDAD.

CONSENTIMIENTO INFORMADO PARA PARTICIPAR EN EL ESTUDIO DE INVESTIGACIÓN

"Prevalencia y factores asociados a infección por VPH en cavidad orofaríngea en estudiantes universitarios".

Xalapa, Ver., a ________de ____________________ del 2023.

Yo ____ estudiante universitario, confirmo que he revisado la hoja informativa para participar en el estudio **"Prevalencia y factores asociados a infección por VPH en cavidad orofaríngea en estudiantes universitarios".**

Comprendo que mi participación es voluntaria y que puedo retirarme del estudio en cualquier momento.

No existiendo ningún inconveniente por mi parte, he decidido ACEPTAR y dar mi consentimiento para participar en este estudio.

Firma del participante

____

Firma del integrante del equipo de investigación

____

Firma de testigo 1 | **Firma de testigo 2**

__________________________ | ______________________________

EN TÉRMINOS DE LA LEY 216 DE PROTECCIÓN DE DATOS PERSONALES EN POSESIÓN DE SUJETOS OBLIGADOS PARA EL ESTADO DE VERACRUZ DE IGNACIO DE LA LLAVE, SE LES INFORMA QUE LAS PERSONAS A CARGO DEL PROYECTO SON RESPONSABLES DEL USO Y TRATAMIENTO QUE SE LE DÉ A LA INFORMACIÓN PERSONAL PROPORCIONADA POR USTED, ASÍ COMO DE SU PROTECCIÓN, COMPROMETIÉNDOSE EN TODO MOMENTO A QUE ESTA SERÁ TRATADA BAJO LAS MÁS ESTRICTAS MEDIDAS DE SEGURIDAD QUE GARANTICEN SU CONFIDENCIALIDAD, INTEGRIDAD Y DISPONIBILIDAD.
