# Supplementary material for: Prevalence and Associated Factors of HPV Infection in the Oropharyngeal Cavity Among University Students in a Southwest Population in Mexico
Source: Diseases. 2025 Dec 31;14(1):16. doi: 10.3390/diseases14010016 (PMC12840128; doi:10.3390/diseases14010016)
Supplement: Supplementary file 1 [file diseases-14-00016-s001.zip › Survey.pdf]

ENCUESTA

Aspectos generales

Sexo	Hombre	Mujer	Edad	_________ años	
Orientación sexual	Heterosexual	Homosexual	Bisexual	Otro	

Aspectos de salud sexual

Edad del primer encuentro sexual	_________ años	Número de parejas sexuales	_____________	¿Has padecido alguna infección de transmisión sexual (ITS)?	Si / No

En caso afirmativo, ¿cuál ITS ha padecido?							
VIH SIDA	Gonorrea	Clamidia	Tricomoniasis	VPH	Sífilis	Herpes Genital	Otro (indica cual)

En caso positivo a VPH ¿Has tenido verrugas genitales?	Si	No	¿Sabes si tu pareja sexual ha padecido alguna infección de transmisión sexual (ITS)?	Si ha padecido / No ha padecido / No sé si ha padecido
¿Has recibido la vacuna contra VPH?	Si / No	En caso afirmativo y si lo recuerdas, ¿a qué edad recibiste la vacuna y en cuántas dosis?	_______años	¿Sabes si tu pareja sexual ha recibido la vacuna contra VPH? — Si la ha recibido / No la ha recibido / No sé si la ha recibido

Hábitos sexuales

¿Has besado en la boca de manera íntima (con lengua) a otra persona?	Si / No	En caso afirmativo	¿A cuántas personas durante el último año? (indica cuántas con número): ____________ ¿A cuántas personas a lo largo de tu vida? (indica cuántas con número): ____________
¿Utilizas preservativo (condón) cuando practicas sexo?	Si / No / En ocasiones	¿Utilizas algún otro método para prevenir la ETS?	No Si ¿cuál? ______________________________
¿Has practicado alguna vez sexo oral (contacto buco-genital) con una pareja sexual?	Si / No	En caso afirmativo	¿Con cuántas personas durante el último año? (indica cuántas con número): ____________ ¿Con cuántas personas a lo largo de tu vida? (indica cuántas con número): ____________ ¿Utilizas preservativo cuándo practicas sexo oral? Si / No / En ocasiones

Hábito tabáquico y alcohólico

¿Has fumado tabaco?	Si / No	En caso afirmativo ¿cómo te clasificas? (señala tu respuesta)	Fumador habitual (1 cigarro o más al día) / Fumador ocasional (Menos de un cigarro al día) / Exfumador (Al menos 8 meses sin fumar)
¿Has tomado alcohol?	Si / No	En caso afirmativo ¿cómo se clasifica? (señala tu respuesta)	Bebedor habitual (Al menos una vez por semana) / Bebedor ocasional (Menos de 10 veces al año) / Exbebedor (Al menos 2 años sin tomar)
¿Consumes algún otro tipo de droga?	Si / No	En caso afirmativo	¿Cuál? (señala tu respuesta): Marihuana / Cocaína / Heroína / Otra

Aspectos sobre el estado general de salud

¿Padeces alguna enfermedad crónica?	Si / No	En caso afirmativo	¿Cuál? (señala tu respuesta): Diabetes / Hipertensión / VIH/sida / Asma / Otra (indica cual) _______
¿Practicas algún deporte o realizas ejercicio?	Sí / No	En caso afirmativo	¿Cuántas horas a la semana? (indica con número): ___________

EN TÉRMINOS DE LA LEY 316 DE PROTECCIÓN DE DATOS PERSONALES EN POSESIÓN DE SUJETOS OBLIGADOS PARA EL ESTADO DE VERACRUZ DE IGNACIO DE LA LLAVE, SE LES INFORMA QUE LAS PERSONAS A CARGO DEL PROYECTO SON RESPONSABLES DEL USO Y TRATAMIENTO QUE SE LE DÉ A LA INFORMACIÓN PERSONAL PROPORCIONADA POR USTED, ASÍ COMO DE SU PROTECCIÓN, COMPROMETIÉNDOSE EN TODO MOMENTO A QUE ESTA SERÁ TRATADA BAJO LAS MÁS ESTRICTAS MEDIDAS DE SEGURIDAD QUE GARANTICEN SU CONFIDENCIALIDAD, INTEGRIDAD Y DISPONIBILIDAD.
